# Effects of Body Image and Self-Concept on the Management of Type 1 Diabetes Mellitus in Adolescents and Young Adults: A Systematic Review

**DOI:** 10.3390/healthcare13121425

**Published:** 2025-06-14

**Authors:** Miguel Garrido-Bueno, Marta Núñez-Sánchez, María Soledad García-Lozano, Javier Fagundo-Rivera, Alba Romero-Alvero, Pablo Fernández-León

**Affiliations:** 1 Centro Universitario de Enfermería Cruz Roja, University of Seville, 41009 Seville, Spain; 2 Department of Nursing, Faculty of Nursing, Physiotherapy and Podiatry, University of Seville, 41009 Seville, Spain; 3 Virgen del Rocío University Hospital, Andalusia Health Service, Junta de Andalucía, 41013 Seville, Spain; 4 Infanta Leonor Hospital, Madrid Health Service, 28031 Madrid, Spain

**Keywords:** type 1 diabetes mellitus, adolescents, young adults, body image, self-concept, glycemic control, disease management

## Abstract

**Background:** Adolescence and young adulthood are critical periods during which psycho-emotional factors can significantly influence disease management and increase the risk of complications. This systematic review aims to examine the impact of body image, self-image, self-perception, and other psycho-emotional variables on the management of type 1 diabetes mellitus (T1DM) in this population. **Methods:** This review follows the Cochrane Handbook, PRISMA 2020 guidelines and the JBI Checklist for Systematic Reviews and Research Syntheses. A comprehensive search was conducted across both general and discipline-specific databases (PubMed, Web of Science, Scopus, Embase, CINAHL, APA PsycInfo, APA PsycArticles) between March and April 2025. The inclusion criteria focused on studies involving adolescents with T1DM that addressed relevant emotional or psychological aspects. Methodological quality was assessed using JBI tools. Data extraction was performed independently by four reviewers, with discrepancies resolved by consensus. A total of 25 studies met the inclusion criteria. **Results:** Body image concerns were found to be highly prevalent among adolescents and young adults with T1DM, and were associated with adverse outcomes such as disordered eating behaviors and suboptimal glycemic control. Gender differences were consistently reported, with adolescent girls and young women displaying greater body dissatisfaction and engaging more frequently in risky weight management practices, including insulin omission. Other factors, such as self-perception, diabetes-specific stress, and identity formation, also played significant roles in treatment adherence and psychosocial adaptation. Notably, this review reveals a lack of interventions specifically designed to address the psychological dimensions of T1DM. **Conclusions:** Body image and self-concept exert a substantial influence on T1DM management in adolescents and young adults, affecting both glycemic outcomes and psychosocial well-being. There is a pressing need for gender-sensitive and developmentally appropriate interventions that address body image, self-concept, and disease acceptance. Future research should prioritize longitudinal designs and the development and evaluation of targeted psycho-emotional support strategies.

## 1. Introduction

Diabetes mellitus comprises a group of chronic metabolic disorders characterized by impaired insulin production and elevated blood glucose levels [1]. According to the 11th edition of the IDF Diabetes Atlas (2025), approximately 589 million adults aged 20–79 are currently living with diabetes, representing 11.1% of the global population within this age group. Projections suggest that this number will increase substantially by 2050 [2].

Among its types, Type 1 diabetes mellitus (T1DM) is insulin-dependent and typically diagnosed in childhood or adolescence. This early onset is associated with heightened vulnerability to both acute and chronic complications—biological, psycho-emotional, and social—during critical developmental periods [3]. It is currently estimated that approximately 9.1 million individuals worldwide are living with T1DM. Despite advances in clinical care and broader access to structured diabetes education, managing T1DM remains a significant challenge, particularly for adolescents and their families [2].

Poor glycemic control in individuals with T1DM can lead to serious biological complications, including microvascular and macrovascular alterations, which require careful the monitoring of parameters such as body mass index and continuous glucose levels [4]. On a psycho-emotional level, adolescents may develop problematic eating behaviors, identity disturbances, and negative perceptions of the disease, often manifested through dissatisfaction with body image, impaired self-concept, and diabetes-related distress [5]. At the social level, family dysfunctions and challenges in relational dynamics may further complicate disease management and everyday habits [6].

Among the different therapeutic approaches, health promotion stands out as a key strategy for empowering individuals and communities to take control of health determinants [7]. Within this framework, patient education—especially led by nurses in both hospital and community settings—is a fundamental intervention for managing chronic conditions. These educational processes aim to support people and their families in handling treatment effectively and preventing avoidable complications [7,8]. They also contribute to improving body image, treatment adherence, coping strategies, and self-care, thereby enhancing quality of life [4].

Existing research has shown that body image problems in people with T1DM are associated with several negative psychosocial and behavioral outcomes. However, most of these studies have focused on adult populations [9]. Few have examined the unique developmental and gender-specific challenges faced by adolescents and young adults living with T1DM, and no systematic review has yet been conducted to synthesize how psycho-emotional variables—specifically body image, self-image, self-perception, and identity—impact disease management in this group. This represents a critical gap in knowledge, as adolescence and young adulthood are periods when individuals are particularly sensitive to issues of physical appearance, peer comparison, autonomy, and emotional regulation, all of which may directly influence their capacity to adhere to complex treatment regimens [10].

Therefore, the aim of this study was to analyze the effects of body image, self-image, self-perception, and other psycho-emotional aspects on the management of T1DM in adolescents and young adults. By identifying key psychological and social influences, this review aims to support the development of more comprehensive and developmentally appropriate interventions that address not only clinical outcomes, but also the emotional and identity-related dimensions of living with T1DM.

## 2. Materials and Methods

This study is a systematic review on the effects of the emotional aspects associated with T1DM on disease management. It was conducted following the Cochrane handbook, the PRISMA 2020 guidelines, and Joanna Briggs Institute (JBI) Checklist for Systematic Reviews and Research Syntheses [11,12,13]. A review protocol was registered in the Open Science Framework (https://doi.org/10.17605/OSF.IO/589RK). No changes were made to the information provided in the protocol.

The inclusion criteria were established based on the characteristics of the studies that could address the following research question formulated using the Population, Exposure, Outcome (PEO) framework [14]: How do emotional aspects associated with T1DM (E) in adolescents and young adults (P) influence disease management (O)?

Accordingly, studies were included if they (1) focused on adolescents (13–18 years) or young adults (19–24 years) diagnosed with T1DM, in accordance with the age classification defined by MeSH terminology, and (2) addressed the emotional, psychological, or psychosocial aspects related to the disease, such as body image, self-concept, or illness perception. No restrictions were placed on publication date, as the topic remains underexplored and limiting by date risked omitting relevant studies. Similarly, no language restrictions were applied; studies published in any language were considered, and translation tools were used as needed, in line with Cochrane recommendations [11].

Exclusion criteria included the following: studies that were not original research articles (e.g., conference abstracts, protocols, literature reviews, or meta-analyses); articles that had been retracted (verified through the Retraction Watch database); and studies that did not report data separately for adolescents and young adults when the sample included broader age ranges. Notably, no studies were excluded based on methodological quality or risk of bias at the eligibility stage; these factors were evaluated post-inclusion using the JBI critical appraisal tools [13].

Information sources were consulted between March and April 2025. These were general databases (PubMed, Web of Science, Scopus, Embase) and specific nursing, psychology, and psychiatry databases (CINAHL, APA PsycInfo, APA PsycArticles). No searches were conducted on websites, organizations, or other additional resources. The search strategy was developed using the MeSH thesaurus descriptors and free terms linked to the research question. These were modified with truncations and joined with Boolean strings: (adolescen* OR teen* OR “young adult*”) AND (“type 1 diabet*” OR “diabetes mellitus type 1” OR “T1DM” OR “T1D” OR “type 1 DM”) AND (“body imag*” OR “self-imag*” OR “self-perce*”) AND (“self-manag*” OR “self-car*” OR “patient educat*” OR “diabetes manag*” OR “glycemic control” OR “treatment adheren*” OR “patient compli*”). It should be noted that NOT was changed to AND NOT in Scopus due to its technical requirements.

The search strategy applied across the selected databases generated the results is presented in Table 1.

The reference selection process was conducted independently by four peer reviewers, based on the predefined eligibility criteria, and was structured in three stages: preprocessing, screening of titles and abstracts, and full-text review [12]. In cases of disagreement, consensus was reached through discussion among reviewers, and the opinion of a fifth reviewer was sought when necessary.

Inter-rater reliability during the study selection process was assessed using Cohen’s Kappa coefficient. The calculation followed the standard formula *κ* = [(Po − Pe)/(1 − Pe)], where *Po* represents the observed agreement and *Pe* represents the agreement expected by chance. Of the 80 full-text articles assessed for eligibility, reviewers agreed on 74, resulting in *Po* = 0.925. The expected chance agreement was *Pe* = 0.375. Substituting this into the formula yields *κ* = [(0.925 − 0.375)/(1 − 0.375)] = (0.55/0.625) = 0.88.

**Figure 1:**
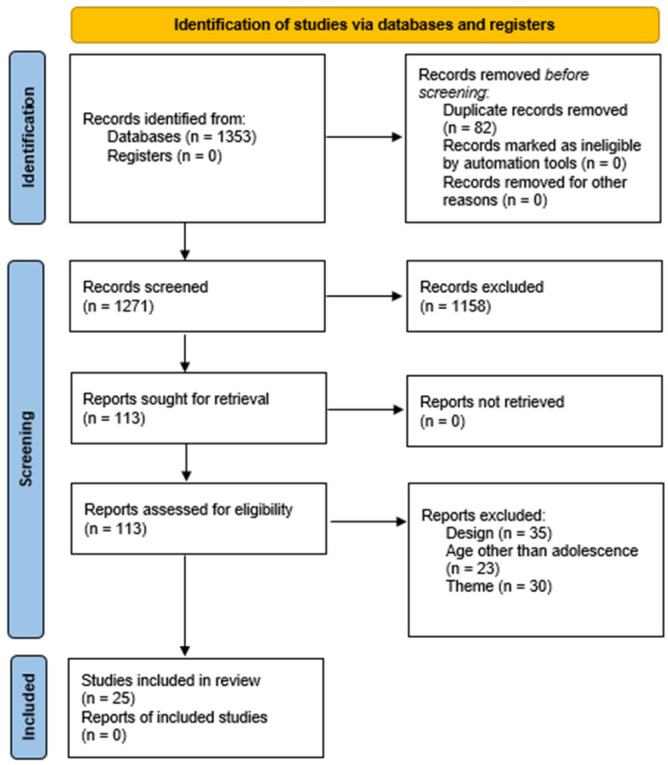
PRISMA flowchart.

The reviewers used Zotero (version 7.0.15) for reference management, and the selection process is visually represented using a PRISMA 2020 flowchart [12] (Figure 1).

Once the literature search was completed and the studies to be included in the present systematic review were selected, the JBI critical appraisal tool [13] was independently applied by four peer reviewers. This process helped identify the potential methodological biases present in the included studies and informed the interpretation of the results, considering the specific characteristics and limitations of each study design. For this review, the JBI checklists for analytical cross-sectional and longitudinal studies [15], randomized controlled trials (RCTs) [16], and qualitative research [17] were used. These critical appraisal checklists are part of the JBI systematic review methodology and are designed to assess the methodological quality of studies and the extent to which potential sources of bias have been addressed in their design, conduct, and analysis. Each checklist consists of a series of questions to be answered with “Yes”, “No”, “Unclear”, or “Not applicable”, followed by an overall appraisal to determine whether the study should be included, excluded, or if further information is required.

Common domains assessed across the checklists include the validity and reliability of exposure or treatment measurement, the identification and management of confounding factors, the validity and reliability of outcome measurement, and the appropriateness of the statistical analysis employed. The checklists are personalized to specific study designs; for example, the analytical cross-sectional checklist includes items on clearly defined inclusion criteria and detailed descriptions of participants and settings, the cohort study checklist addresses group similarity and follow-up strategies, the RCT checklist focuses on the randomization procedures and blinding of participants and personnel, and the qualitative research checklist assesses data analysis, synthesis, and presentation of findings, as well as transparency in the reporting of the methodological approach [13,15,16,17].

Disagreements between reviewers were resolved through discussions, and the opinion of a fifth reviewer was sought when consensus could not be reached. Inter-rater reliability was high (*κ* = 0.90), indicating almost perfect agreement according to the Landis and Koch classification [18].

The data extraction process was carried out independently by four peer reviewers using Microsoft Excel (version 2016). In cases of disagreement, consensus was reached through discussion among reviewers, and a fifth reviewer was consulted when necessary. Inter-rater reliability was high (*κ* = 0.92), indicating almost perfect agreement according to the Landis and Koch classification [18]. The data collected and recorded for each study included the following: (a) reference, authorship, year, and region of publication; (b) study design, intervention characteristics, variables examined, sample size, and JBI appraisal score; (c) study objective; and (d) findings related to the psycho-emotional aspects of T1DM. Confounding information was intentionally excluded from the analysis. In this review, one example of such confounding was the failure to disaggregate outcomes specific to adolescents when results were reported for broader age ranges that included, but did not isolate, adolescent participants.

A narrative synthesis was conducted using a conceptual domain-based grouping method. These domains were developed inductively based on recurring patterns and central topics across the studies, and they were subsequently refined through team discussions and consensus. The final domains used to structure the results were as follows: (1) the characteristics of the included studies; (2) the prevalence and correlates of body image concerns and self-perception; (3) the impact of body image concerns and self-perception on glycemic control and T1DM management; (4) T1DM-specific stress, identity, and coping mechanisms; and (5) intervention strategies and gaps related to the psycho-emotional aspects of T1DM management.

## 3. Results

A total of 25 studies were chosen for this systematic review (Table 2).

### 3.1. Methods and General Characteristics of the Studies

The included studies were conducted across various countries, including the United States [19,23,31,32,41], Italy [34,39,40], Belgium [35,42], Australia [27,36], Germany [28,30], the United Kingdom [21,43], the Netherlands [25], Malaysia [38], Sweden [20], Canada [33], Taiwan [22], Egypt [37], Palestine [26], South Korea [29], and Ethiopia [24].

The 25 studies were published across several years, starting in 1999 [21] and ending with the most recent publication in 2024 [42]. The study with the largest sample size was Vanderhaegen et al. [42] (*n* = 558), while the smallest sample size was found in Jeong et al. [29] (*n* = 14).

Regarding the quantitative design, this research includes twelve cross-sectional [19,22,24,25,26,27,32,34,37,40,41,43], eight longitudinal studies [21,28,30,31,33,35,39,42], and one randomized controlled trial [20]. On the other hand, three qualitative studies have been included [23,29,38]. Finally, the research includes one mixed-methods study [36].

The methodological quality assessment using JBI tools revealed overall moderate-to-high rigor across the included studies. Most cross-sectional studies scored between 7 and 8 out of 8, with several (e.g., [32,36,40]) achieving perfect scores. Longitudinal studies showed more variability, with scores ranging from 6 to 10 out of 11; Reference [35] stood out with the highest score, while others had notable limitations. The only randomized controlled trial [20] scored 9 out of 13, suggesting good quality. All of the qualitative studies included scored 10 out of 10, indicating excellent methodological consistency (Supplementary Tables S1–S4).

### 3.2. Prevalence, Correlates, and Gender Differences in Body Image Concerns, Emotional Responses, and Self-Perception

Several studies have explored the frequency and reasons behind body image concerns in adolescents and young adults with T1DM [25,32,39]. A significant interaction was found between gender and body mass index on body dissatisfaction, as well as between gender and body dissatisfaction on the desire for thinness [32]. Similarly, it was observed that most young people with T1DM tended to perceive themselves as thinner than they were and desired to be even thinner, and that body dissatisfaction was a unique predictor of problematic eating behaviors [39]. This desire for thinness and body dissatisfaction are confirmed in cross-sectional studies, such as the one by Eilander et al. [25], where almost half of adolescents with T1DM reported concerns about their body image and weight, and body dissatisfaction was significantly related to disturbed eating behaviors.

The influence of gender is consistently highlighted [19,26,32,38]. In addition to its significant interaction with other variables on body dissatisfaction and desire for thinness [32], other studies found that body image was a frequent concern among adolescents with T1DM, leading them to reduce food intake [38], and even found that a significant percentage of adolescent girls with T1DM, compared to those without the pathology, omitted or reduced their insulin dose to lose weight and were more likely to report body dissatisfaction [19]. This finding is reinforced by the study by Olmsted et al. [33], which identified that concern about weight and figure, as well as self-esteem based on appearance, were strong predictors of the development of eating disorders in adolescent girls with T1DM.

Other associated factors include concern about medical devices [23] and the greater evaluative salience of appearance in younger adults with T1DM [27]. In addition, other studies observed that adolescents with T1DM with higher body mass indexes tended to manifest disordered attitudes towards eating, with a greater impact on women [43], and these studies also confirmed a high prevalence of eating disorders in adolescents with T1DM, being more frequent in girls and significantly associated with body image problems [40]. More recent studies found a high prevalence of body dissatisfaction associated with eating disorders [24] and reported a significant proportion of adolescents with T1DM with poor body image [37].

### 3.3. Impact of Body Image Concerns and Self-Perception on Glycemic Control and T1DM Management

Several studies have shown a connection between body image concerns and diabetes management [25,28,32]. Elevated scores on the bulimia subscale were significant predictors of Hb A1c test [32]. Similarly, adolescents with eating disorders were found to have significantly higher Hb A1c levels and lower self-care confidence, showing impaired disease management [25]. At the family level, it was also found that, in single adolescents, a worse perception of body image mediated the relationship between a negative family climate and impaired glycemic control [28].

Insulin restriction, as a behavior associated with body image concerns, also negatively impacts glycemic control [21,27]. Younger adults who reported restricting insulin to control weight showed higher Hb A1c [27], with women being more affected [21].

Self-perception also plays a relevant role on glycemic control and T1DM management [22,26,31,40,43]. Studies found a negative correlation between perceived health and Hb A1c, as well as between a sense of coherence and Hb A1c in adolescents with T1DM, suggesting that better self-perception and sense of coherence are associated with better glycemic control [26] and that higher Hb A1c was observed in overweight/obese participants who were also at higher risk of disordered eating [31]. Other studies indicated that young people with T1DM who reported disordered attitudes towards eating tended to have worse glycemic control [40,43], so much so that greater variability in Hb A1c levels was associated with a higher risk of eating disorders in this population [22].

### 3.4. T1DM-Specific Stress, Identity, and Coping Mechanisms

Other studies have explored the psychosocial burden of living with this condition, particularly in relation to identity [23,35,42]. Adolescents with T1DM reported feelings of frustration and exhaustion due to the constant monitoring, even leading to the denial or refusal of treatment as a coping mechanism. However, a process of acceptance and adaptation over time was also observed [23]. Other studies found that rejection of the T1DM identity negatively predicted treatment adherence, and both rejection and absorption were associated with higher levels of disease-specific stress [35]. Stress and elevated Hb A1c levels were also found to predict the increased absorption of the disease identity [42].

Regarding coping mechanisms, a positive correlation was found between perceived health and sense of coherence in adolescents with T1DM [26], as well as a higher positive self-concept in those adolescents and young adults with optimal glycemic control [30]. Another study highlighted the strong desire of young adults with T1DM to be recognized as individuals beyond their diagnosis, experiencing frustration when their identity was reduced to their medical condition and expressing a longing for “normality” that sometimes led to self-care avoidance and feelings of embarrassment when managing diabetes in public [29].

### 3.5. Interventions Strategies and Gaps in the Psycho-Emotional Aspects of T1DM Management

Regarding interventions, one based on the Guided Self-Determination-Young model improved glycemic control after 12 months in adolescents initiating continuous subcutaneous insulin infusion [20]. Another intervention based on insulin pump therapy was associated with a lower prevalence of eating disorders after several months of study [31]. These studies suggest that patient- and technology-centered interventions could have benefits regarding both metabolic and eating behavior aspects.

## 4. Discussion

This systematic review examines the impact of body image and self-concept on the treatment of T1DM, particularly among adolescents and young adults. The findings suggest that these populations are especially vulnerable to challenges related to body image perception, DEBs, and the integration of disease identity. These factors negatively influence glycemic control and psychosocial adjustment.

### 4.1. Identity Development and T1DM Management

Adolescence and early adulthood are periods of increased sensitivity due to asynchronous neurocognitive development, increased social demands, and the emergence of mental health problems [44]. During these stages, the construction of self-concept and the integration of a chronic disease into one’s personal identity are critical for the effective management of T1DM [23]. Longitudinal studies indicate that diabetes-related identity tends to evolve through phases of rejection, absorption, and acceptance. Rejection of this identity has been associated with a lower level of adherence to treatment and a higher level of diabetes-related distress, while acceptance and a sense of personal growth are linked to better adherence and reduced stress.

Managing T1DM imposes a considerable psychosocial burden, particularly when the self-definition process overlaps with a crucial stage of identity formation [45]. Qualitative studies have shown that young people with T1DM perceive the condition as a persistent disruption of daily life and a continuous source of stress, affecting not only their quality of life, but also their self-image and perception of their body.

The visibility of medical devices, such as insulin pumps or continuous glucose monitors, can contribute to discomfort and social stigmatization, particularly in school or social settings. This discomfort can lead to avoidant behaviors, including temporarily removing devices or refraining from using them in public, which compromises disease management and reinforces a sense of “abnormality” that negatively affects self-perception [46].

### 4.2. Eating Behaviors and Glycemic Control

One of the most concerning patterns identified in this review is the intentional restriction or omission of insulin as a weight control strategy, predominantly reported among young women with T1DM. This behavior, which is clinically dangerous and psychologically distressing, is associated with poorer glycemic control and a greater risk of microvascular complications [47]. The motivations that motivate them often include body dissatisfaction, social pressure, and a desire for physical normality, emphasizing the need for targeted interventions that address these psychosocial aspects.

The relationship between body image, DEBs, and glycemic control is complex and multifaceted [40]. Several studies have confirmed that body dissatisfaction is a significant predictor of DEBs in adolescents with T1DM. These behaviors, which range from dietary restriction to binge eating, have detrimental effects on glycemic outcomes. Potential mechanisms include emotional dysregulation, low self-esteem, and social pressures with respect to physical appearance. Furthermore, recent research suggests that variability in Hb A1c levels, in addition to average levels alone, is also linked to an increased risk of eating disorders, pointing to a dimension of metabolic instability that is associated with psychological distress.

Although most of the included studies focused on the psychosocial factors influencing eating behaviors and glycemic control in adolescents with T1DM, it is important to situate these behaviors within the broader context of global nutritional transitions. While the findings from Ahmad and Yuasa are not specific to T1DM, they highlight how rapid nutritional shifts—characterized by sedentary lifestyles and poor-quality diets—can contribute to metabolic risk and unhealthy weight gain beginning early in life. Such environments may exacerbate challenges in glycemic control for youth with T1DM, particularly when disordered eating patterns and body dissatisfaction are present [48].

### 4.3. Gender Differences

The findings of this review consistently highlight the critical role of gender in the manifestation of concerns about body image and the prevalence of DEBs among people with T1DM [49]. Adolescent girls and young women with T1DM exhibit higher levels of body dissatisfaction, a stronger desire for thinness, and a greater tendency to engage in risky weight management behaviors, such as deliberately reducing or skipping insulin doses. This pattern has been documented in multiple quantitative and qualitative studies, including both cross-sectional and longitudinal designs.

From a sociocultural perspective, these gender differences can be understood within the framework of normative beauty ideals, which disproportionately impact women, especially during adolescence, a developmental stage marked by intensified social pressure to conform to esthetic standards [10]. Internalization of these ideals can create a profound conflict between optimal management of diabetes, which often involves visible medical devices and treatment-induced changes in body weight, and the desire to conform to the social standards of an acceptable or desirable body [50].

This analysis gains additional relevance when contextualized within the 2030 Agenda for Sustainable Development of the United Nations, particularly Goal 3 (Good Health and Well-being) and Goal 5 (Gender Equality) [51]. Neglecting the influence of gender in the management of chronic diseases such as T1DM not only jeopardizes the physical and mental health of women, but also perpetuates structural disparities in access, quality of care, and health equity [52,53].

### 4.4. Influence of Family and Social Environment

Social and familial environment also plays a critical role in these dynamics. A negative family environment, as well as the perceived stigma related to the use of medical devices, has been associated with poorer body image and lower self-concept, which may indirectly affect glycemic control [8,54]. On the contrary, a positive perception of health and a high sense of coherence have been associated with better metabolic outcomes, suggesting that interventions targeting these factors could prove beneficial.

Self-esteem, particularly in relation to physical appearance, has emerged as a significant risk factor for the development of body dissatisfaction in adolescents with T1DM [55]. Studies included in this review report that low self-esteem correlates with increased weight-related concerns and negative body image, which, in turn, can hamper treatment adherence.

Additionally, longitudinal studies have shown that a negative family climate characterized by conflict, emotional unavailability, or excessive control over treatment can directly affect perceptions of body image in adolescents. In such cases, body image functions as a mediator variable between family dynamics and glycemic control, further highlighting its clinical relevance in the comprehensive treatment of T1DM.

### 4.5. Limitations and Strengths

Despite the valuable insights provided by this review, it is important to recognize several limitations inherent in the included studies. A large proportion of the evidence is derived from cross-sectional designs, which limits the ability to establish causal relationships between body image concerns and diabetes management outcomes. Many studies also relied on self-reported data, which are subject to recall bias and social desirability effects. Furthermore, longitudinal studies remain scarce, impeding our complete comprehension of the long-term psychosocial trajectories associated with T1DM in adolescence and young adulthood.

In addition to methodological limitations, broader contextual factors should be considered. Cultural differences in body image ideals, health beliefs, and gender norms may influence how adolescents perceive themselves and manage their condition. These variables are often underreported or not accounted for in the literature, potentially limiting the generalizability of findings across different sociocultural contexts. In this way, future research should prioritize longitudinal, culturally sensitive designs that can capture the complex, evolving relationship between self-concept and chronic illness management.

Despite these limitations, this review has several strengths. It is the first to systematically synthesize evidence on the intersection between body image, self-concept, and disease management specifically in adolescents and young adults with T1DM. The utilization of a comprehensive search strategy, adherence to rigorous methodological standards (PRISMA, Cochrane Handbook, and JBI Checklist), and the inclusion of both qualitative and quantitative evidence provide a robust and multidimensional comprehension of the topic. This integrative perspective enhances the clinical relevance of the findings and supports the design of more targeted and developmentally appropriate interventions.

### 4.6. Future Perspectives

#### 4.6.1. The Role of Technology in Psychological Support

The increasing use of advanced technologies in the management of T1DM, such as continuous subcutaneous insulin infusion and continuous glucose monitoring, has significantly transformed the experience of living with this condition. A peer-reviewed study suggests that these technologies do not negatively impact body image, at least in the short term. However, it is imperative to conduct longitudinal research focused on vulnerable subgroups to fully understand their influence on body perception, adherence to treatment, and mental health.

In this context, the emerging use of tools and digital platforms based on artificial intelligence (AI) presents a unique opportunity to monitor, predict, and intervene in a personalized manner when signs of psychological risk arise, such as the development of DEBs or the rejection of treatment [56]. The integration of AI with clinical and psychosocial data could enable automated early detection, thus improving clinical response and facilitating more accurate and timely interventions [57].

#### 4.6.2. Media and Social Media Influence

Although the impact of media and social networks on body image has been widely documented in the general population [58], its specific influence on young individuals with T1DM is still lacking evidence. Digital platforms, although potentially a source of esthetic pressure and social comparison, can also serve as educational and health promotion tools if strategically used [59].

Digital campaigns, influencers living with T1DM, virtual support groups, and positive narratives on social media could play a meaningful role in reducing stigma, normalizing the use of medical devices, and improving self-concept among young people with diabetes [53,60].

#### 4.6.3. The Importance of Qualitative Approaches

Given the prevalence of quantitative studies, qualitative research emerges as an essential tool for capturing the subjective experience of living with T1DM, particularly in relation to body image, self-concept, and coping strategies [61]. The qualitative studies reviewed revealed powerful narratives that involve frustration, shame, the desire for normalcy, and avoidance behaviors that shape the daily lives of many individuals with this condition.

These studies give attention to personal experiences, help identify psychosocial barriers often overlooked in biomedical models, and shed light on the emotional impact of factors such as the use of visible medical devices or body-related social commentary. Qualitative approaches are also critical for exploring the experiences of less represented groups (e.g., men with DEBs, LGBT individuals, ethnic minorities) who are frequently excluded from large-scale population studies [62].

Despite significant advances in the technological and metabolic management of T1DM, psycho-emotional care remains an often-neglected area, especially in relation to body image and self-perception. This review indicates that, although some preliminary efforts have been made, there is a notable lack of interventions specifically targeting the psychological dimensions of T1DM.

Although patient-centered models, such as Guided Self-Determination Young, have shown promise in improving glycemic control and adherence to treatment, their impact on body image, self-esteem, and DEB prevention has not been thoroughly evaluated [20]. Based on these findings, there is a clear need to foster the development and evaluation of interventions that directly and explicitly address concerns related to physical appearance, self-concept, and acceptance of disease.

Potential strategies include adapted cognitive behavioral therapy for people with T1DM, acceptance and commitment therapy focusing on the integration of disease identity, educational programs that combine body image literacy with diabetes management, and the promotion of peer emotional support networks [63]. These interventions should be gender and developmentally sensitive and should preferably be evaluated using randomized controlled trials with long-term follow-ups [64].

#### 4.6.4. Cross-Cultural Perspectives

As this review incorporates studies conducted in diverse sociocultural settings—including the USA, Italy, Belgium, Malaysia, Egypt, and Palestine—the need for cross-cultural research becomes evident. Such research should explore how social norms, cultural values, and healthcare systems shape body image, self-esteem, and diabetes-related behaviors.

Concerns related to body image and weight, as well as coping strategies, are not universally experienced; stigma can take different forms depending on the cultural context, and local beauty ideals can exert varying levels of pressure [10]. Therefore, comparative studies are essential to identifying cultural similarities and differences in disease experience and to adapting clinical interventions accordingly.

A cross-cultural lens is also crucial for the development of culturally sensitive assessment tools and relevant educational programs that reflect local realities, avoiding the uncritical application of models developed in high-income countries without appropriate adaptation or validation [65].

## 5. Conclusions

This systematic review aimed to analyze the effects of body image, self-image, self-perception, and other psycho-emotional aspects on the management of T1DM in adolescents and young adults. The evidence gathered from 25 studies reveals the multifactorial and interrelated impact of these variables on both glycemic control and the psychosocial well-being of young individuals with T1DM.

Concerns about body image were found to be highly prevalent, particularly among adolescent girls and young women, and were frequently associated with DEBs, including insulin omission as a method of weight control. These behaviors, in turn, contribute to poorer glycemic control and increased risk of complications. Self-concept and self-perception also emerged as critical factors influencing adherence to treatment and adjustment to the disease, with low self-esteem and negative self-image correlating with worse clinical and emotional outcomes.

Moreover, identity issues, especially difficulties in integrating the disease into one’s self-concept, were linked to denial, treatment nonadherence, and emotional distress. Adolescents who demonstrated higher levels of disease acceptance tended to show better adherence and improved metabolic outcomes, whereas those who internalized stigma or rejected their illness experienced higher psychological burden.

These findings emphasize the need for clinical approaches that go beyond metabolic control, addressing the emotional, cognitive, and social dimensions of diabetes management. Interventions should be gender-sensitive and developmentally personalized, incorporating body image work, emotional regulation strategies, and self-concept strengthening into routine diabetes care. Nurses, psychologists, and interdisciplinary teams have a key role in designing and delivering these integrative interventions.

Finally, future research should focus on longitudinal studies to better understand the causal relationships and temporal dynamics between psycho-emotional factors and diabetes outcomes, as well as focusing on evaluating the efficacy of psychosocial interventions that target body image, self-perception, and disease acceptance in this vulnerable population.

## Figures and Tables

**Table 1:** Search strategy applied in each database.

Database	Search Strategy	Search Date	Outcomes	Selected
PubMed	(“adolescen*”[All Fields] OR “teen*”[All Fields] OR “young adult*”[All Fields]) AND (“type 1 diabet*”[All Fields] OR “diabetes mellitus type 1”[All Fields] OR “T1DM”[All Fields] OR “T1D”[All Fields] OR “type 1 DM”[All Fields]) AND (“body imag*”[All Fields] OR “self imag*”[All Fields] OR “self perce*”[All Fields]) AND (“self manag*”[All Fields] OR “self car*”[All Fields] OR “patient educat*”[All Fields] OR “diabetes manag*”[All Fields] OR “glycemic control”[All Fields] OR “treatment adheren*”[All Fields] OR “patient compli*”[All Fields])	01/03/25	57	5
WOS	(adolescen* OR teen* OR “young adult*”) AND (“type 1 diabet*” OR “diabetes mellitus type 1” OR “T1DM” OR “T1D” OR “type 1 DM”) AND (“body imag*” OR “self-imag*” OR “self-perce*”) AND (“self-manag*” OR “self-car*” OR “patient educat*” OR “diabetes manag*” OR “glycemic control” OR “treatment adheren*” OR “patient compli*”) (Topic)	07/03/25	82	1
CINAHL	(adolescen* OR teen* OR “young adult*”) AND (“type 1 diabet*” OR “diabetes mellitus type 1” OR “T1DM” OR “T1D” OR “type 1 DM”) AND (“body imag*” OR “self-imag*” OR “self-perce*”) AND (“self-manag*” OR “self-car*” OR “patient educat*” OR “diabetes manag*” OR “glycemic control” OR “treatment adheren*” OR “patient compli*”)	13/03/25	35	3
Scopus		20/03/25	73	3
Embase	(adolescen* OR teen* OR ‘young adult*’) AND (‘type 1 diabet*’ OR ‘diabetes mellitus type 1′/exp OR ‘diabetes mellitus type 1′ OR ‘t1dm’/exp OR ‘t1dm’ OR ‘t1d’ OR ‘type 1 dm’) AND (‘body imag*’ OR ‘self-imag*’ OR ‘self-perce*’) AND (‘self-manag*’ OR ‘self-car*’ OR ‘patient educat*’ OR ‘diabetes manag*’ OR ‘glycemic control’/exp OR ‘glycemic control’ OR ‘treatment adheren*’ OR ‘patient compli*’)	25/03/25	82	6
APA PsycInfo	“(adolescen* OR teen* OR (“young adult” OR “young adulthood” OR “young adults”)) AND (“type 1 diabet*” OR “diabetes mellitus type 1” OR “T1DM” OR “T1D” OR “type 1 DM”) AND ((“body image” OR “body images” OR “body imaging”) OR “self-imag*” OR “self-perce*”) AND (“self-manag*” OR “self-car*” OR (“patient education”) OR (“diabetes management”) OR “glycemic control” OR (“treatment adherence”) OR (“patient compliance”))”	01/04/25	972	4
APA PsycArticles		02/04/25	52	3
Total			1353	25

**Table 2:** Summary of selected articles.

Author, Year, Reference and Region	Methodology: 1. Design 2. Intervention 3. Variables of interest 4. Sample 5. JBI Score	Aim	Main Results and Conclusions
Ackard et al. [19] 2008 United States	Cross-sectional study. Without intervention. *Type 1 diabetes mellitus (T1DM) group*: 143 adolescents (73 men and 70 women) who participated in the AHEAD study. The average age was 15.3 years. *Comparison group* (general population): 4746 youth (2377 men, 2357 women, and 12 of unspecified sex) who participated in Project EAT. The mean age was 14.9 years. JBI: 5/8.	To compare the prevalence of disordered eating and body dissatisfaction among adolescents with T1DM and a sample of young people from the general population.	Adolescents with T1DM did not have a higher risk of unhealthy weight-control behaviors or weight dissatisfaction compared to other adolescents. In fact, they reported less weight dissatisfaction and were less likely to engage in some unhealthy weight-control behaviors. Furthermore, they consumed meals (breakfast, lunch, and dinner) more frequently than their peers without diabetes. However, despite medical supervision, the study identified a concerning prevalence of insulin manipulation as a means of weight control among youth with T1DM. Specifically, a significant percentage of girls (10.3%) and a small percentage of boys (1.4%) with diabetes reported skipping insulin doses or taking less than prescribed to lose weight or prevent weight gain. This finding is of great concern given the serious medical complications associated with inadequate glycemic control. Further analysis revealed that adolescents with diabetes who manipulated insulin were more likely to report body dissatisfaction than those with diabetes who did not. Approximately 45.5% of youth with diabetes who manipulated insulin were very dissatisfied or dissatisfied with their weight, compared to 10.9% of those who did not. No significant differences in Body Mass Index (BMI) were found between these two groups.
Brorsson et al. [20] 2019 Sweden	Randomized clinical trial. The intervention group attended seven group sessions over a five-month period, using the GSD-Y model. The control group received standard care. Variables: Glycated hemoglobin (Hb A1c) at 6 and 12 months, self-perceived health, health-related quality of life, family conflicts, self-efficacy, and use of continuous glucose monitoring. The sample consisted of *n* = 41 women and *n* = 28 men. Their ages ranged from 12 to 17.99 years. JBI: 9/13.	To evaluate whether the person-centered education and communication model, called Guided Self-Determination-Young (GSD-Y), improves glycemic control, self-rated health, health-related quality of life, reduces diabetes-related family conflicts, and improves self-efficacy in adolescents who initiate continuous subcutaneous insulin infusion (CSII) therapy with their parents.	When adjusted for sex and family conflict, there were no differences in Hb A1c between the intervention and control groups at enrollment; 8.4% (SD 0.9%) vs. 8.8% (SD 1.2%) (68 [SD 10.2] vs. 73 [SD 12.7] mmol/mol, *p* = 0.06); or at 6 months: 7.6% (SD 0.9%) vs. 8.0% (SD 1.0%) (60 [SD 9.8] vs. 64 [SD 10.4] mmol/mol, *p* = 0.19). At 12 months, a difference was detected between the groups: 7.8% (SD 1.1%) vs. 8.6% (SD 1.1%) (62 (SD 11.4) vs. 70 (SD 12.1) mmol/mol, *p* = 0.009). When analyses were performed for boys and girls separately and adjusted for family conflict, a difference was detected for boys after 12 months: 7.4% (SD 0.9%) vs. 8.3% (SD 1.1%) (57.0 (SD 10.1) vs. 67.3 (SD 11.5) mmol/mol, *p* = 0.019). In boys, an intervention effect was identified after 6 months: −1.0% (SE 0.3%) (−11.1 [SE 3.7] mmol/mol, *p* = 0.004) and 12 months: −1.0% (SE 0.3%) (−11.2 [SE 3.5] mmol/mol, *p* = 0.002). In girls, a difference was only identified in the control group after 6 months: −0.8% (SE 0.3%) (−8.2 [SE 3.7] mmol/mol, *p* = 0.029). The level of family conflicts was 25 (25, 27) at the beginning (*n* = 66), 25 (24, 28) at 6 months (*n* = 48) and 24 (23, 27) at 12 months (*n* = 39). At baseline, the intervention group perceived more family conflicts related to diabetes (intervention 25 vs. control 22, *p* = 0.027), but there were no differences were observed at six months (intervention 24 vs. control 23, *p* = 0.258) or twelve months (intervention 22 vs. control 24, *p* = 0.417). At baseline, the control group had a higher total score on the Swe-DES “Readiness to Change” domain and a lower perceived physical burden of diabetes. There were no differences in health, health-related quality of life, or diabetes burden between the groups at 6 or 12 months. There were no significant differences in Hb A1c values between adolescents who used CGM and those who did not use it at any time during the study.
Bryden et al. [21] 1999 UK	Longitudinal observational study. Quantitative approach with analysis of data collected over 8 years. Without intervention Eating habits, weight, glycemic control, and insulin use were monitored in adolescents with T1DM. Anthropometric variables: Weight, height, body mass index (BMI). Psychological variables: Concern about body shape and weight, dietary restriction, and presence of eating disorders (measured with the Eating Disorder Examination). Insulin Use: Record of intentional omission or reduction in insulin for weight control. Glycemic control: Glycosylated hemoglobin (Hb A1c) levels. Diabetic complications: Urine albumin/creatinine ratio and presence of microvascular complications (retinopathy, nephropathy, hypertension). Initial population: 76 adolescents (43 men and 33 women) with T1DM, aged 11–18 years, assessed in 1989–1990 and diagnosed with diabetes at least one year before the start of the study. Follow-up: 65 of the original 76 participants (86%) were reassessed between 1997 and 1998, when they were aged 20–28 years (one of the non-interviewees died of diabetic ketoacidosis, another died of severe mental disability due to a severe hypoglycemic episode). JBI: 6/11.	To examine the relationship between dietary habits, insulin misuse, changes in body weight, and their impact on glycemic control and diabetic complications in adolescents with T1DM over eight years. To explore the relationship between eating disorders/insulin misuse and glycemic control, as well as the presence of diabetic complications.	Height, weight, and BMI; formula: [(observed population mean)/population SD], appropriate for the subject’s sex and age. Initial and follow-up assessment of eating disorder features was performed using the Eating Disorders Examination. The interview was adapted to distinguish between behaviors necessary for diabetes management, such as avoiding sugary foods for glycemic control, and those attributable to an eating disorder, such as extreme dietary restriction for figure and weight control. At each assessment, subjects were asked whether they had ever reduced or omitted insulin use to lose weight. At the follow-up assessment, past and present misuse and its probable duration were determined. *Weight gain*: Both men and women increased their weight and BMI from adolescence to adulthood, with women being overweight at both assessments. *Weight concern*: Increased significantly in both sexes, reflected in higher levels of dietary restriction. Women showed significantly greater concern about weight and shape at follow-up than during adolescence. Of the women who expressed greater weight concern at follow-up, 72% had a BMI of one standard deviation greater than at baseline. Men showed a similar pattern of greater concern at follow-up, although at much lower levels than women at both assessments. Weight change in men, but not in women, correlated with change in level of dietary restraint (rs = 0.42; *p* = 0.008). *Eating disorders*: No cases of anorexia or bulimia nervosa were identified, but mild forms of eating disorders were observed, especially in women. Six subjects (one man and five women) from either the initial assessment (*n* = 3) or the follow-up interview (*n* = 3) met criteria for eating disorder not otherwise specified (EDNOS), either with markedly abnormal behavior (*n* = 3), such as recurrent self-induced vomiting or laxative use, or with abnormal importance given to shape and weight (*n* = 3). One woman was classified as having EDNOS at both assessments. She was diagnosed with bulimia nervosa between the completion of the initial and follow-up interviews, and has been receiving treatment for several years. Insulin misuse: 30% of women admitted to intentionally reducing or omitting insulin to control their weight. The duration of misuse varied considerably, with a minimum of 3 months and an average of 2 years. Forty-five percent of women with microvascular complications had engaged in this practice. Four women who had abused insulin were among the six subjects classified as having a clinical eating disorder (CED). No women admitted to insulin abuse at the follow-up evaluation. No men admitted to deliberate insulin abuse in any of the evaluations. *Glycemic control*: No relationship was observed between subjects with EDNOS and glycemic control, nor was there any difference between the glycemic control of subjects with EDNOS with abnormal behavior (vomiting, laxative use) and those with abnormal psychiatric behavior. The mean Hb A1c of the ten women who admitted to intentional insulin misuse was worse than that of the remaining women, both at baseline (10.3 ± 1.1 vs. 9.5 ± 2.0) and at follow-up (9.7 ± 1.8 vs. 9.2 ± 1.9), although none of the differences were statistically significant. *Risk of complications:* Insulin omission was associated with poor glycemic control and could contribute to the development of diabetic complications. Of the women who developed microvascular complications, five (46%) deliberately misused insulin (one was diagnosed with EDNOS at baseline and one with EDNOS during follow-up). Two women had laser-treated proliferative retinopathy, two had nephropathy, and one had both laser-treated proliferative retinopathy and nephropathy. No significant associations were observed between baseline eating disorders or insulin misuse and the development of diabetic complications. An overall mean Hb A1c over the 8 year period between baseline and follow-up shows significantly worse long-term glycemic control in these subjects.
Chou et al. [22] 2023 Taiwan	Quantitative. Without intervention. *n* = 110 patients with T1DM and *n* = 32 with Type 2 diabetes mellitus (T2DM). All were receiving insulin treatment at a tertiary care center. Age, mean (SD): T1DM 17.70 years (5.05); T2DM 16.19 years (4.14) JBI: 7/8.	To investigate the clinical and behavioral interrelationship between eating disorders and insulin restriction and their association with psychological health.	Patients with T1DM tended to be less concerned about body image and used medication less as a method of weight control. Regarding the univariate regression analysis, it was observed that the CR of the TFEQ-R21 scale was (OR = 2.37, [95% CI: 1.04–5.40]), body image (OR = 2.07, [95% CI: 1.25–3.44]), diet (OR = 6.48, [95% CI: 2.50–16.77]), and excessive exercise (OR = 8.42, [95% CI: 1.51–46.85]), which were associated with an mSCOFF score of 2 or higher. In contrast, only Hb A1c-SD (OR = 2.18, [95% CI 1.07–4.42]), body image (OR = 1.83, [95% CI 1.05–3.20]), and diet (OR = 4.74, [95% CI 1.70–13.23]) were associated with an mSCOFF score of 2 or higher. Hierarchical regression analysis showed a lower standard deviation of Hb A1c (odds ratio = 2.1 8, [95% CI: 1.07–4.42]), body image (1.83, [1.05–3.20]), and diet (4.74, [1.70–13.23]) associated with disordered eating behavior (DEB) and clinical and behavioral correlations between eating disorders and insulin restriction (ED/IR). Furthermore, ED/IR behavior was associated with anxiety (1.17 [1.08–1.27]) and depression (1.12 [1.03–1.22]). Across the different forms, it was observed that mSCOFF scores were consistently associated with depression and anxiety according to the HADS questionnaire, even after controlling for clinical and behavioral parameters. In the fully adjusted model, an mSCOFF score of 2 or higher was associated with a 17% increased OR for anxiety and a 12% increased OR for depression according to the HADS questionnaire.
Commissariat et al. [23] 2016 USA	Qualitative. Without intervention. Hb A1c, a total of 5 questions about living with diabetes and self-image with diabetes *n* = 40. Forty-seven percent of the participants were women (*n* = 19). Participants ranged in age from 13 to 30 years. The mean age of the sample was 16.15 ± 1.89 years. JBI: 10/10.	Exploring the incorporation of T1DM into self-identity among adolescents.	One of the main themes is diabetes as a burden on daily life. Within this theme, adolescents’ express feelings of frustration and exhaustion due to the constant need to monitor their health. “It’s like going through an epic journey where you know you’re not going to reach a finish line, but everybody tells you it’s about trying. It’s difficult to look yourself in the face in the morning if you know you haven’t done what you need to do, and so I feel like diabetes is associated with a big guilt trip, and it’s life-long, and it sucks, hard” (19-year-old female). In some cases, adolescents choose denial or rejection of treatment as a way to cope with the disease. “I think I almost sabotage myself sometimes because I want to get back at it or rebel. I think it’s interesting what the mind does sometimes. I sabotage myself. Like too eating much or not bolusing the way I should. I’m only harming myself, not anyone else. It’s just a really bad habit that I formed” (17-year-old female). Concerns about body image are also present, as some adolescents have been the subject of negative comments or ridicule due to the medical devices they use. “In public, I didn’t like checking my blood sugar and taking needles because someone would always ask, ‘What is that, what’re you doing?’ I had an incident before where I was actually made fun of, for having a really old pump. Then I took my pump off and didn’t wear it for almost 2 years because I was insecure about it” (17-year-old female). However, other adolescents describe a process of acceptance and adaptation over time. “Now I’m at a place where it’s just I’ve accepted it. It’s something I know I’m going to live with for the rest of my life, I know there’s nothing I can do except learn to take care of it and be healthy about it… you just have to learn the steps necessary for you to be a healthy member of society and for you to learn that this is what you have to deal with” (18-year-old female).
Daniel et al. [24] 2023 Ethiopia	Cross-sectional study. Without intervention. The study focused on the prevalence of disordered eating behaviors and how they related to body shape dissatisfaction in a group of adolescents with T1DM receiving medical care in hospitals in Addis Ababa. The study sample consisted of a total of 395 adolescents with diabetes. Regarding age, participants ranged in age from 10 to 19 years. The median (±IQR) age of participants was 15 (±11 to 19) years, and the majority (approximately 61%) were in their early teens (11–15 years). 52.2% were women and 47.8% were men. JBI: 7/8.	To assess the magnitude of DEBs and their relationship with body shape dissatisfaction among adolescents with diabetes in Addis Ababa, Ethiopia.	The results revealed a high prevalence of BDDs in this population, reaching 43.3% in the past 30 days. Furthermore, 20.3% of adolescents reported dissatisfaction with their body shape. One of the most relevant findings was the significant association between body dissatisfaction and the presence of BDDs. Adolescents dissatisfied with their body were 2.2 times more likely to develop DEBs. In addition to body image, other factors significantly associated with BDDs included having a family history of diabetes mellitus, being in late adolescence (16–19 years), experiencing diabetic complications, and being overweight. Regarding the types of eating behaviors observed, a higher prevalence of non-purging behaviors, such as skipping meals and avoiding glycemic control, was identified, rather than more aggressive practices such as self-induced vomiting. Furthermore, scores on the DEPS-R scale were significantly higher among adolescents with body dissatisfaction, overweight, and those from higher-income families. However, no significant differences were found in these scores between males and females. The study’s findings highlight that DEBs constitute a significant health problem among adolescents with T1DM in this context. Furthermore, factors such as negative body image, overweight, family background, and health complications associated with diabetes significantly contribute to the development of these behaviors.
Eilander et al. [25] 2017 Netherlands	Quantitative, descriptive, cross-sectional. Without intervention. Age, age at onset, duration of diabetes, family structure, type of treatment, Hb A1c *n* = 103. The mean age was 13.5 years (SD = 1.49); *n* = 53, 51.5% of participants were girls. JBI: 6/8.	To explore the prevalence of DEBs and associated ‘yellow flags’.	In total, 80.4% (*n* = 83) of participants used an insulin pump as part of their treatment. The mean Hb A1c was 8.0% (SD = 0.64) [range: 5.1–15.8%]. The mean age-adjusted BMI (BMIz) was 0.64 (SD = 1.0). The mean age at diabetes onset was 7.0 years (SD = 3.9), and the duration of diabetes was 6.5 years (SD = 3.8). In total, 46.5% of adolescents with T1DM reported concerns about their body image and weight. Eight percent of participants exceeded the clinical threshold for DEBs. Adolescents with DEBs had higher Hb A1c levels, with a statistically significant difference (*p* = 0.004). These adolescents were also found to have lower confidence in their self-care and diabetes management (*p* = 0.015), which is associated with a reduction in their quality of life (*p* = 0.007). Adolescents with DEBs showed significant impairment in diabetes management (*p* < 0.001). Body dissatisfaction was related to the presence of DEBs (*p* < 0.001), while BMI was not. Dieting frequency was found to be a significant risk factor for the development of DEBs (*p* = 0.001).
Elissa et al. [26] 2020 Palestine	Quantitative, cross-sectional study. Without intervention. *n* = 300 healthy children aged 8–18 from six primary, secondary and high schools in the north, south and central West Bank. JBI: 7/8.	To measure perceived health status in adolescents and sense of coherence (SOC) in children with T1DM and to examine possible correlations between sociodemographic and medical characteristics.	Regarding self-perceived health status, men showed a higher level compared to women, M = 84.0 (SD = 11.44) versus 75.21 (SD = 17.86), *p* = 0.008, on the generic scale. The study found a positive correlation between self-perceived health status and SOC, directly proportional; higher self-perceived health status was associated with higher SOC (*p* < 0.001). Furthermore, a negative correlation was found between perceived health status and Hb A1c (*p* = 0.003). The higher the perceived health, the lower the Hb A1c, i.e., the better the glycemic control. The relationship between SOC and Hb A1c was found to be negative (*p* = 0.012), where a higher SOC was associated with a lower Hb A1c.
Gawlik et al. [27] 2015 Australia	Quantitative, descriptive, cross-sectional. Without intervention. Age, diabetes duration, quality of life, salience, Hb A1c, body image, adjustment to diabetes *n* = 177. The mean age of participants was 36.32 years (SD = 11.33, *n* = 176), with a range of 18 to 68 years. JBI: 5/8.	To study and evaluate the associations between the appearance investment component of body image, age, quality of life, and self-reported metabolic control.	The mean duration of diabetes was 18.39 years (SD = 11.15, *n* = 176), with a range from 1 to 48 years. The mean self-reported Hb A1c was 7.84% (SD = 1.63, *n* = 169), with a range from 4.5 to 14.7%. The results showed that self-evaluative salience (the degree to which appearance influences self-worth) was higher in younger participants (*p* < 0.001), those with a lower quality of life (*p* < 0.001), and those with better metabolic control (*p* < 0.001). On the other hand, motivational salience (the degree of attention and effort devoted to appearance) was not significantly associated with any of the study variables. Participants who reported restricting insulin to control their weight showed higher self-evaluative salience (*p* < 0.001), lower quality of life (*p* < 0.001), and worse levels of metabolic control (higher Hb A1c, *p* < 0.05). The prevalence of insulin restriction in the sample was 21%. The study also confirmed that hypoglycemia and hyperglycemia were associated with a worse quality of life (*p* < 0.01).
Hartl et al. [28] 2015 Germany	Quantitative, descriptive, longitudinal. Without intervention. Age, age of diagnosis, family type, perceived family climate, body image, relationship status *n* = 109. The mean age of participants was 15.84 years (SD = 1.44), with a range of 13 to 19 years. The sample consisted of *n* = 51 (46.8%) girls and *n* = 58 (53.2%) boys. JBI: 9/11.	To assess whether body image mediates longitudinal links between family climate and changes in glycemic control in adolescents.	The majority of participants came from families with two biological parents (*n* = 94, 86%). Among adolescents who were not in a romantic relationship, body image mediated the relationship between family climate and changes in glycemic control over time. A worse family climate at age 16 was associated with worse body image perception at the same year (β = 0.56, *p* < 0.001), which in turn predicted deterioration in glycemic control between ages 16 and 17 (β = −0.43, *p* < 0.05). The direct relationship between family climate and glycemic control was no longer significant when body image was included in the analysis (indirect effect: β = −0.24, SE = 0.09, *p* = 0.007). In adolescents who were in a romantic relationship, no significant associations were found between family climate and changes in glycemic control (β = −0.02, *p* > 0.05), nor was there a mediating effect of body image (indirect effect: β = 0.00, SE = 0.004, *p* > 0.05). Furthermore, the correlation between body image and glycemic control at age 17 was negative and significant only in single adolescents (r = −0.54, *p* < 0.001), whereas it was not observed in those with a partner (r = −0.02, *p* > 0.05).
Jeong et al. [29] 2018 South Korea	Descriptive qualitative study using focus groups. Two focus group sessions were conducted with a total of 14 participants. A semi-structured conversation format with an interview guide and verbal probing techniques were used to obtain participants’ responses. *n* = 14 participants. Regarding gender, there were nine women (64.3%) and five men (35.7%). The participants’ ages ranged from 20 to 34 years (mean = 26.5 years; SD = 4.5 years). JBI: 10/10.	To explore health-related stigma among young adults with T1DM using descriptive qualitative methods in focus groups.	The results revealed five main themes. First, participants expressed a strong desire to be recognized as people and not just as individuals with a disease, feeling frustration when their identity was reduced to their medical condition. They also expressed a longing for normality and to be equal to their peers without T1DM, which sometimes led them to avoid self-monitoring their glucose and insulin. Embarrassment about managing diabetes in public caused stress and promoted concealment of their self-care practices. Another important finding was anger and distress stemming from stereotypes and lack of knowledge about T1DM, along with distrust from family members and healthcare professionals regarding their ability to manage the disease independently. In terms of body image and self-image, stigma negatively affected participants’ self-perception. The feeling of being seen only because of their diagnosis and low self-esteem associated with social devaluation contributed to a negative identity. The desire to “be like everyone else” reflected how diabetes generated feelings of difference compared to their peers.
Luyckx et al. [30] 2009 Germany	Eight-phase longitudinal cohort study. Family climate (at times 1–4) and self-concept (at times 1–4 and 6) were assessed. Times 1–4 covered adolescence, with mean ages of 14 to 17 years, respectively. Times 5–8 covered emerging adulthood, with mean ages of 21 to 25 years, respectively. Glycemic control, general family climate, and self-concept. *n* = 72 people with T1DM. At time 1, the start of the study, the mean age was 13.72 years (SD = 1.46). The age range covered adolescence (mean ages 14–17 years) and emerging adulthood (mean ages 21–25 years). 37 women and 35 men. JBI: 7/11.	To determine developmental patterns of glycemic control in young people with T1DM throughout adolescence and emerging adulthood, and to assess relationships with overall family climate and self-concept.	Throughout adolescence and emerging adulthood, the study identified three distinct trajectories of glycemic control in people with T1DM: optimal, moderate, and impaired. The data showed that those with optimal glycemic control tended to maintain a higher positive self-concept, especially at key points in the study. Conversely, those with impaired control scored lowest on this psychological dimension. From late adolescence onward, these trajectories began to differentiate more clearly, intensifying during emerging adulthood. Both family climate and self-concept in middle and late adolescence were observed to act as psychosocial markers associated with these trajectories. Self-concept was assessed using the Offer Self-Image Questionnaire, which includes dimensions of the psychological self—such as impulse control, emotional tone, and body image—and the coping self. Although body image was not analyzed independently, it was considered an essential component of overall self-concept. These findings reinforce previous research indicating a significant relationship between self-concept and the evolution of glycemic control. They also highlight the importance of fostering a positive self-concept in adolescents with T1DM, as this could translate into sustained benefits for disease management. In particular, it has been observed that adolescents may experience lower self-esteem and greater distress related to their condition, factors possibly linked to self-image, although this dimension has not been explored in detail. Ultimately, this research highlights the role of self-concept as a relevant psychosocial factor in the treatment and progression of T1DM, and suggests that strengthening this dimension during adolescence could contribute to better long-term glycemic control.
Markowitz et al. [31] 2013 USA	Quantitative, control study. Without intervention. Sample: At baseline there were 43 young people (45% women) with T1DM, although the analyses include only the 37 participants, aged 10–17 years, who completed the Diabetes Specific Eating Problems Score (DSPS-R) at all three time points. JBI: 6/11.	To investigate the DSPS-R, a validated measure of risk for both diabetes-specific and general eating disorders.	Those enrolled in the study had a mean Hb A1c level of 8.3–1.3% (68%–14.5 mmol/mol) at baseline. DEPS-R scores decreased over time (*p* = 0.01). The overall rate of high-risk eating disorders was low. Overweight/obese youth experienced more DEBs than normal-weight participants. DEPS-R scores correlated with body mass index z score at all three time points and with Hb A1c at 1 and 6 months. Hb A1c did not change significantly and was higher in overweight/obese participants than in normal-weight participants (8.7% [72 mmol/mol] vs. 7.8% [62 mmol/mol]; *p* = 0.005). One-third of participants decreased A1c by 0.5% (5 mmol/mol), one-third increased it by ±0.5% (5 mmol/mol), and one-third remained within 0.5% (5 mmol/mol). There was a significant difference in DEPS-R scores between men and women, with women scoring higher (women, 11.0 [25th–75th percentile, 8.0–15.0]; men, 5.0 [25th–75th percentile, 2.5–11.2]; *p* = 0.04). Furthermore, those with overweight/obesity obtained significantly higher DEPS-R scores than those with normal weight.
Meltzer et al. [32] 2001 USA	Quantitative, descriptive, cross-sectional. Without intervention. Eating Disorders Inventory; Body dissatisfaction, desire for thinness, and bulimia, Hb A1c, BMI *n* = 152. The sample consisted of 54% boys and 46% girls. The mean age of the children was 14.45 years (SD = 1.99), while the mean disease duration was 6.08 years (SD = 3.49). JBI: 8/8.	To examine the relationship between disordered eating attitudes and behaviors, BMI, and glycemic control in adolescents with T1DM.	Regarding pubertal development, 5.4% of the participants were in Tanner stage 1, 10.9% in stage 2, 14.0% in stage 3, 27.1% in stage 4, and 42.6% in stage 5. The mean HbA₁c was 9.04% (SD = 1.67), while the mean body mass index (BMI) was 22.02 kg/m^2^ (SD = 4.36). Regarding body dissatisfaction, the interaction between gender and BMI was found to be significant (β = 1.08, *p* = 0.03), suggesting that the relationship between BMI and body dissatisfaction varies by gender; however, neither gender (β = −0.54, *p* = 0.17) nor BMI alone (β = −0.11, *p* = 0.64) were significant predictors, with the model explaining 32.8% of the variance (r^2^ = 0.328, F = 18.24). In relation to bulimia, the interaction between age and gender was a significant predictor (β = 0.59, *p* < 0.001), indicating that the influence of age on bulimia symptoms varies by gender, while neither age (β = −0.15, *p* = 0.11) nor gender alone (β = −0.17, *p* = 0.16) were significant predictors, with the model explaining 20.5% of the variance (r^2^ = 0.205, F = 10.05). For thinness desire, the interaction between gender and body dissatisfaction was significant (β = 0.72, *p* = 0.01), suggesting that the impact of body dissatisfaction on thinness desire differs by gender, while neither gender (β = 0.12, *p* = 0.17) nor body dissatisfaction alone (β = −0.05, *p* = 0.85) were significant, with the model explaining 55.6% of the variance (r^2^ = 0.556, F = 53.82). Regarding BMI, bulimia symptoms were a significant predictor (β = 0.21, *p* = 0.03), with the model explaining 5.7% of the variance (r^2^ = 0.057, F = 6.41). Finally, in glycemic control measured by HbA₁c, disease duration (β = 0.25, *p* = 0.01), high scores on the bulimia subscale (β = 0.19, *p* = 0.05), and obesity (β = 0.16, *p* = 0.09) were significant predictors, with the model explaining 12.2% of the variance (r^2^ = 0.122, F = 4.55).
Olmsted et al. [33] 2008 Canada	Prospective cohort study. Body mass index (BMI) percentile, concern about weight and figure, global and appearance-based self-esteem, and depression. *n* = 126 girls with T1DM. At the start of the study, the participants were between 9 and 13 years old. JBI: 8/11.	To identify predictors of the onset of DEBs in adolescents with T1DM.	The study revealed that certain psychological and physical factors are closely related to the onset of DEBs, accounting for 48.2% of its occurrence. This integrative model includes concerns about weight and shape, self-esteem—both global and focused on physical appearance—BMI percentile, and depressive symptoms. When analyzing each factor separately, concerns about weight and shape, as well as self-esteem related to physical appearance, were found to be the strongest predictors of the development of DEBs, explaining 21.0% and 20.0% of the variance, respectively. Global self-esteem was also significant, although with a lesser impact, explaining 4.4% of the variance. The results showed that girls who, one or two years before starting DEBs, had a high BMI, greater body concerns, impaired self-esteem—especially related to appearance—and depressive symptoms were more likely to develop these types of behaviors.
Peducci et al. [34] 2018 Italy	Quantitative. Without intervention. *n* = 85 preadolescents and adolescents with T1DM (60% women). 60% were adolescents of mean age 13.4 ± 4.8 years. The mean age of onset of T1DM was 7.1 ± 4.0 years. JBI: 7/8.	The objective was to investigate eating patterns and eating behaviors in the adolescent population with T1DM.	The study highlighted that 43 of the patients (50.5%) reported binge eating episodes. In total, 20% of the binges were deliberate, and 17.6% suppressed or decreased their insulin dose. These were more common in patients who binge ate(χ^2^ = 4.58; *p* < 0.03). Binge eating was more common in girls than in boys. Of the 51 girls, 21.5% (or 11 girls) reported skipping insulin doses to lose weight. Of these, 10 were overweight. In contrast, only 2 boys manipulated insulin doses to do so (*χ*^2^ = 3.87; *p* = 0.049). Regarding Hb A1c values, there were no significant differences between girls who reported binge eating (7.8 ± 0.6%) and those who did not (7.3 ± 1.2%).
Rassart et al. [35] 2021 Belgium	Quantitative, descriptive, longitudinal (3 years). Without intervention. Age, sex, disease duration, treatment type, disease identity, diabetes-related distress, treatment adherence, Hb A1c. *n* = 276. Participants ranged in age from 14 to 15 years (mean = 19 years). 19.54% were female. JBI: 10/11.	To examine developmental trajectories of illness identity and potential associations between illness identity and diabetes-specific functioning.	Results indicated small but significant increases in acceptance (slope = 0.05, *p* < 0.01) and absorption (slope = 0.03, *p* < 0.05) and a decrease in rejection (slope = −0.08, *p* < 0.001). Refusal was found to negatively predict treatment adherence one year later (standardized coefficient = −0.08, *p* < 0.05), whereas enrichment was found to positively predict it (standardized coefficient = 0.06, *p* < 0.05). Furthermore, treatment adherence subsequently predicted higher levels of enrichment (standardized coefficient = 0.05, *p* < 0.05) and lower absorption (standardized coefficient = −0.05, *p* < 0.05). Both refusal and absorption predicted higher levels of diabetes-specific stress 1 year later (refusal: standardized coefficient = 0.09, *p* < 0.05; absorption: standardized coefficient = 0.10, *p* < 0.01). Stress and elevated Hb A1c levels were also found to predict increased absorption 1 year later (stress: standardized coefficient = 0.11, *p* < 0.01; Hb A1c: standardized coefficient = 0.06, *p* < 0.05).
Robertson et al. [36] 2020 Australia	Mixed-methods study. Without intervention. Sexual behavior and activity, body image, and anxiety, both in people with T1DM who use continuous insulin infusion (CSII) and continuous glucose monitoring (CGM) technologies, and in those who do not. *n* = 285 participants with T1DM. Mean age: 34.5 ± 13.3 years, with an age range of 16 to 60 years. 53% (*n* = 152) of respondents were women, 46% (*n* = 132) were men. JBI: 8/8; 10/10.	To explore the impact of external diabetes technologies on sexual behavior and activity, body image, and anxiety in adopters and non-adopters of these devices.	Despite common concerns about the visibility of technologies such as CSII, especially among women who report feeling more self-conscious about having devices attached to their bodies, research found no clear evidence that these tools have a negative impact on body image. The study revealed no significant differences in body dissatisfaction—measured using the Stunkard Figure Rating Scales—between those who use technologies such as CSII or CGM and those who do not. This absence of differences persisted even when analyzing the data by gender and age. This suggests that the decision not to adopt these technologies is not necessarily related to greater body image concerns or associated anxiety, as is sometimes assumed. In other words, although some patients with T1DM prefer to avoid visible devices for personal reasons, this preference does not appear to be linked to greater body dissatisfaction.
Salah et al. [37] 2022 Egypt	Qualitative, cross-sectional study. Without intervention. *n* = 60 adolescents with T1DM, patients of the Pediatric Diabetes Clinic at Ain Shams University Children’s Hospital between December 2020 and March 2021. The mean age of the studied adolescents with T1D was 13.35 ± 3.28 years. They were 35 females (58.3%) and 25 males (41.7%). JBI: 7/8.	The objective of the study is to compare DEB (prevalent comorbidity among adolescents with T1DM) in adolescents with T1DM receiving different therapies and correlate it with body image, glycemic control, and depressive symptoms.	Of the 60 adolescents, 14 had a poor body image (23.3%); of the remaining 46, 42 had a moderate body image (70%), and only 4 had a good body image (6.7%). Of all of them, 22 adolescents with T1DM had depression (36.7%). Another data that the study demonstrated was that no significant relationship was found between socioeconomic level and DEB (*p* = 0.634).
Sien et al. [38] 2020 Malaysia	Qualitative. Without intervention. Gender, age, relatives with T1DM. *n* = 15. Most participants were female (*n* = 11, 73.3%), while males accounted for a smaller proportion (*n* = 4, 26.7%). The majority were aged 13–15 years (*n* = 10, 66.7%), followed by those in the 16–18 age group (*n* = 4, 26.7%), and a smaller number in the 10–12 age group (*n* = 1, 6.7%). JBI: 10/10.	To determine the factors of eating disorders in adolescents with T1DM.	Regarding family history of T1DM, only *n* = 1, 6.7% of participants reported a family history of the disease, while the vast majority *n* = 14, 93.3% had no family history. One of the main themes is pressure, which comes from different sources such as school life, family, and friends. Adolescents mention that their academic load and school activities make it difficult to eat regularly, leading them to skip meals. “(Skip lunch time) because the schedule at school is quite busy, there’s a lot to do, so there’s no time to queue and buy food…” (021, female, 13 years old). Social pressure also influences their relationship with food, as criticism or comments about their food intake affect their behavior. “Because when eating with friends, they say ‘Why are you eating so much? Don’t be like this.’ But when I’m in front of the locker, I just eat how much I want and it feels like eating alone is much better…” (038, female, 14 years old). The family is also a source of pressure when they insist, they eat a certain way. “Because I think she (mother) always eat three times a day and maybe she does not understand that I’m not hungry and do not want to eat. Grandma as well. (They will tell me) you will lose weight (and that’s) unhealthy…” (007, female, 13 years old). Another theme identified is the physiological factor. Several adolescents report that their lack of appetite or fatigue influences their eating behavior, leading them to skip meals. “I skipped the morning meal because I’m not hungry, so I don’t even need to eat if I’m not hungry…” (007, female, 13 years old). Body image is a common concern, especially among adolescent girls, who report that their dissatisfaction with their bodies leads them to reduce their food intake to avoid weight gain. “Because I keep gaining weight after (I was) diagnosed with diabetes, so I just wanted my weight to be 50 kg and I am a female, so I wanted to be beautiful…” (021, female, 13 years old). Another relevant issue is the low adherence to insulin intake and dietary control. Some participants acknowledge that they struggle to control their food intake and that, despite knowing they should moderate it, they are unable to do so. “My weight gained because I keep eating too much and I just cannot stop eating…” (038, female, 14 years old). In some cases, adolescents view insulin administration as annoying and do not give it the importance it deserves. “Sometimes I forgot to take insulin…” (021, female, 13 years old). Fear is also a factor influencing the development of eating disorders. “The doctor said it’s very dangerous if I keep gaining weight, so I just wanted my weight to be 40 kg, like that only…” (038, female, 14 years old).
Troncone et al. [39] 2018 Italy	Quantitative, descriptive, longitudinal (5 years). Without intervention. Age, Hb A1c, age at onset, BMI, body image perception and satisfaction (Collin body silhouette), problematic eating behaviors (PEBEQ). *n* = 8. At baseline, the mean age of participants was 7.86 years (SD = 1.5) with a range of 5.1 to 10.06 years, while at follow-up the mean increased to 12.7 years (SD = 1.49) with a range of 10.07 to 15.08 years. JBI: 8/11.	To examine changes over a five-year period in body image accuracy and dissatisfaction, as well as relationships with DEBs, in young patients with T1DM.	HbA1c values remained relatively stable between baseline (8.16 SD = 0.94) and follow-up (7.92 SD = 1.08). The age at onset of the condition averaged 4.69 years (SD = 2.247), and disease duration increased from 2.9 years (SD = 2.44) at baseline to 7.4 years (SD = 2.23) at follow-up. A significant increase in BMI was observed, from −0.19 (SD = 1.26) at baseline to 0.97 (SD = 0.76, *p* < 0.001) at follow-up. Regarding weight category, the percentage of underweight participants decreased from 37.3% at baseline to 0% at follow-up, while the percentage of normal weight participants increased from 31.3% to 29.9%. An increase was seen in the proportion of overweight participants, rising from 25.4% to 43.3%, and in obesity, rising from 6% to 26.8%. At baseline and follow-up, the majority of subjects, over 70%, selected a perceived weight figure category that was thinner than their actual weight. Initially, 50.7% of participants chose an ideal figure category that was thinner than their perceived weight, regardless of their actual BMI. However, at follow-up, the majority of subjects, 52.3%, showed no discrepancy between their ideal weight and their perceived weight. An analysis of differences between mean perceived/ideal body image category and BMI weight category showed that participants tended to perceive themselves as thinner than they actually were at both baseline (t(66) = 5.131, *p* < 0.001) and follow-up (t(66) = 16.046, *p* < 0.001), and that they desired to be thinner than their actual body size at both measurements (baseline t(66) = −3.081, *p ≤* 0.01; follow-up t(66) = 15.893, *p* < 0.001). No significant differences were found between baseline and follow-up assessments in body size estimation accuracy (F(1,66) = 1.415, *p* = 0.24) or body image dissatisfaction (F(1,66) = 1.499, *p* = 0.22), even when gender differences were considered in both comparisons (gender × misperception score interaction: F(1,65) = 0.576, *p* = 0.45; gender × FID score interaction: F(1,65) = 1.466, *p* = 0.23). No significant differences were found between men and women on the total PEBEQ score (t(65) = −0.341, *p* = 0.7), although body dissatisfaction was found to uniquely predict the score on this questionnaire (β = 0.272, *p* = 0.02), while body image misperception did not show a significant relationship with DEBs (β = 0.019, *p* = 0.88).
Troncone et al. [40] 2020 Italy	Cross-sectional study. Without intervention. The variables of interest were body image problems, eating disorder symptoms in parents, and emotional and behavioral difficulties related to the presence of DEBs in a group of adolescents with T1DM. *n* = 200 adolescents with T1DM. Participants ranged in age from 13.02 to 18.05 years, with a mean age of 15.24 years (standard deviation = 1.45). There were 102 boys and 98 girls. JBI: 8/8.	To examine the associations of DEBs with body image problems, parental eating disorder symptoms, and emotional and behavioral problems in adolescents with T1DM.	The main results of this study indicate that a significant proportion of Italian adolescents with T1DM present DEBs, with a prevalence of 36.5%, being more frequent in girls. Adolescents with T1DM and DEBs showed worse metabolic control, higher BMI levels, greater eating disorder symptoms, and significantly more body image problems, as well as greater emotional and behavioral difficulties compared to those without DEBs. When comparing adolescents with T1DM with a normative sample, both boys and girls with T1DM reported more eating disorder symptoms, greater internalization of social attractiveness ideals, and increased emotional and behavioral problems. A hierarchical regression analysis identified pressure to conform to social norms regarding body image and externalizing symptoms as significant predictors of BDDs in both sexes.
Tse et al. [41] 2012 USA	Quantitative, descriptive, cross-sectional. Without intervention Age, diet reports, Hb A1c, BMI, eating problems with diabetes, attitude toward eating, treatment adherence. *n* = 151. Age range: 8–18 years old although the current investigation was limited to those higher or equal to 13 years old. JBI: 7/8.	To expand current knowledge on DEBs in adolescents with T1DM by examining the relationship of DEBs with dietary intake and attitudes toward healthy eating.	Forty-eight percent of participants were female. Adolescents at risk for eating disorders showed a higher prevalence of overweight/obesity compared to the low-risk group (59.1% vs. 40.9%, *p* = 0.01). They also had lower diet quality (HEI-2005: 45.9% vs. 53.7%, *p* = 0.003) and higher intakes of total fat (38.2% vs. 34.4% of total energy, *p* = 0.01) and saturated fat (14.0% vs. 12.2%, *p* = 0.007). No significant differences were observed in total energy intake (*p* = 0.48) or in the distribution of macronutrients from carbohydrates (*p* = 0.11) and proteins (*p* = 0.15). Regarding eating attitudes, the at-risk group showed lower self-efficacy for healthy eating (3.5 vs. 3.9, *p* = 0.005), greater perceived barriers to healthy eating (2.4 vs. 1.9, *p* < 0.001), and higher negative outcome expectations (2.9 vs. 2.1, *p* < 0.001). They also reported lower satisfaction with their diet (3.9 vs. 4.3, *p* = 0.004). Regarding diabetes management, the at-risk group showed lower adherence to treatment in both adolescent self-report (65.4 vs. 76.2, *p* < 0.001) and parental report (69.1 vs. 77.0, *p* = 0.002). They also monitored their glucose less frequently (3.2 vs. 4.7 times/day, *p* = 0.002) and had higher glycosylated hemoglobin levels (Hb A1c: 10.1% vs. 8.6%, *p* < 0.001).
Vanderhaegen et al. [42] 2024 Belgium	Quantitative, descriptive, longitudinal. Without intervention. Personal identity trajectory classes, levels of illness integration. *n* = 558. The sample consisted of *n* = 257 (46.06%) men and *n* = 301 (53.94%) women. The mean age was 18.85 years (SD = 3.24). JBI: 7/11.	To examine whether youth with T1DM belonging to different personal identity trajectories developed four dimensions of disease identity differently.	The mean age at diabetes diagnosis was 11.22 years (SD = 5.52). The majority of participants used injections (*n* = 437, 78.74%), while *n* = 118 (21.26%) used an insulin pump. An increase in acceptance over time was found (slope = 0.05, *p* < 0.01) and a decrease in rejection (slope = −0.08, *p* < 0.001). Absorption showed a slight increase (slope = 0.03, *p* < 0.05). Treatment adherence one year later was negatively predicted by rejection (β = −0.08, *p* < 0.05) and positively predicted by enrichment (β = 0.06, *p* < 0.05). In turn, greater treatment adherence predicted an increase in the feeling of enrichment over time (β = 0.05, *p* < 0.05). Both absorption and rejection in illness identity were found to predict higher levels of diabetes-specific distress one year later (rejection: β = 0.09, *p* < 0.05; absorption: β = 0.10, *p* < 0.01). Furthermore, distress and poor glycemic control predicted increased absorption in illness identity one year later (distress: β = 0.11, *p* < 0.01; Hb A1c: β = 0.06, *p* < 0.05).
Wilson et al. [43] 2015 England	Cross-sectional study. Without intervention. Eating disorder symptoms/attitudes/behaviors in youth with T1DM, along with diabetes-specific risk factors (BMI, glycemic control, diabetes-related family conflict) and general risk factors (dysfunctional perfectionism, self-esteem, gender, and parental eating disorder symptoms). *n* = 50 young people between the ages of 14 and 16 with T1DM. Of these participants, 30 were women and 20 were men. JBI: 7/8.	To examine risk factors for eating disorders in young people with T1DM.	Research shows that young people with T1DM who exhibit disordered eating attitudes and behaviors tend to have a higher BMI, poorer glycemic control, lower self-esteem, and greater family conflict. Women are more affected, especially in terms of body dissatisfaction. It is concluded that, in addition to BMI and blood glucose, it is key to consider self-esteem and family environment in assessment and therapeutic intervention. The findings revealed that adolescent girls with a higher body mass index (BMI-z) tended to report more disordered eating attitudes and behaviors than their male peers, suggesting that the impact of weight on body dissatisfaction is greater in women. In parallel, self-esteem was analyzed using the Rosenberg Self-Esteem Scale. The results showed that young people with disturbed eating attitudes and behaviors had significantly lower self-esteem than those without such disorders. This low self-esteem was not only more common in those with eating disorders, but also served as a clear marker to distinguish these young people from the rest. Taken together, the data demonstrate how body image and self-image are deeply intertwined in the context of T1DM, affecting women more intensely and highlighting the importance of self-esteem as a key factor in the detection and understanding of eating disorders.

Note. Authors’ own elaboration.

## Data Availability

All data are available within this article and Supplementary Files.

## Supplementary Materials

The following supporting information can be downloaded at: https://www.mdpi.com/article/10.3390/healthcare13121425/s1, Table S1. Scores of analytical cross-sectional studies; Table S2. Scores of longitudinal studies; Table S3. Score of randomized controlled trial; Table S4. Scores of qualitative research.

## Abbreviations

The following abbreviations are used in this manuscript (displayed in alphabetical order):

AI	Artificial Intelligence
DEB	Disordered eating behavior
HbA1c	Hemoglobin A1c
JBI	Joanna Briggs Institute
LGBT	Lesbian, Gay, Bisexual, Transgender
PEO	Person, Exposure, Outcome
PRISMA	Preferred Reporting Items for Systematic Reviews and Meta-Analyses
T1DM	Type 1 Diabetes Mellitus
