# Supplementary material for: Effects of Body Image and Self-Concept on the Management of Type 1 Diabetes Mellitus in Adolescents and Young Adults: A Systematic Review
Source: Healthcare (Basel). 2025 Jun 14;13(12):1425. doi: 10.3390/healthcare13121425 (PMC12193616; doi:10.3390/healthcare13121425)
Supplement: Supplementary file 1 [file healthcare-13-01425-s001.zip › healthcare-3638440-supplementary.pdf]

SUPPLEMENTARY MATERIAL

Table S1. Scores of analytical cross-sectional studies.

**Studies**	**JBI**	**Inclusion criteria are clearly defined**	**The participants and the environment are described in detail**	**Exposure was validly and reliably measured**	**The criterion used to measure the condition was objective**	**Confounding factors were identified**	**Strategies for dealing with confounding factors**	**Results measured in a valid and reliable way**	**Appropriate statistical analysis was used**
Ackard et al. [19]	5/8	YES	YES	NO	NO	YES	YES	NO	YES
Chou et al. [22]	7/8	YES	YES	YES	NO	YES	YES	YES	YES
Daniel et al. [24]	7/8	YES	YES	YES	NO	YES	YES	YES	YES
Eilander et al. [25]	6/8	YES	YES	YES	YES	NO	NO	YES	YES
Elissa et al. [26]	7/8	YES	YES	YES	NO	YES	YES	YES	YES
Gawlik et al. [27]	5/8	YES	YES	YES	NO	YES	NO	NO	YES
Meltzer et al. [32]	8/8	YES	YES	YES	YES	YES	YES	YES	YES
Peducci et al. [34]	7/8	YES	YES	YES	YES	YES	NO	YES	YES
Robertson et al. [36]	8/8	YES	YES	YES	YES	YES	YES	YES	YES
Salah et al. [37]	7/8	YES	YES	YES	NO	YES	YES	YES	YES
Troncone et al. [39]	8/8	YES	YES	YES	YES	YES	YES	YES	YES
Tse et al. [41]	7/8	YES	NO	YES	YES	YES	YES	YES	YES
Wilson et al. [43]	7/8	YES	YES	YES	YES	YES	NO	YES	YES

Note. Studies presented by authors' surname alphabetical order. JBI: Joanna Briggs Institute.

Table S2. Scores of longitudinal studies.

Studies	JBI	The two groups were similar and recruited from the same population	The exposures were measured similarly	The exposure was measured in a valid and reliable way	Confounding factors were identified	Strategies to deal with confounding factors were stated	The groups/participants were free of the outcome at the start of the study	The outcomes were measured in a valid and reliable way	The follow-up time was reported and was sufficient	Follow-up was complete, and if not, the reasons were described and explored	Strategies to address incomplete follow-up were utilized	Appropriate statistical analysis was used
Bryden et al. [21]	6/11	NO	YES	YES	NO	NO	NO	YES	YES	YES	NO	YES
Hartl et al. [28]	9/11	NO	YES	YES	YES	YES	YES	YES	YES	NO	YES	YES
Luyckx et al. [30]	7/11	NO	YES	YES	YES	NO	NO	YES	YES	NO	YES	YES
Markowitz et al. [31]	6/11	NO	YES	YES	YES	NO	NO	YES	YES	NO	NO	YES
Olmsted et al. [33]	8/11	NO	YES	YES	YES	YES	YES	YES	YES	NO	NO	YES
Rassart et al. [35]	10/11	YES	YES	YES	YES	YES	NO	YES	YES	YES	YES	YES
Troncone et al. [39]	8/11	NO	NO	YES	YES	YES	NO	YES	YES	YES	YES	YES
Vanderhaegen et al. [42]	7/11	NO	YES	YES	YES	NO	NO	YES	YES	NO	YES	YES

Note. Studies presented by authors' surname alphabetical order. JBI: Joanna Briggs Institute.

Table S3. Score of randomised controlled trial.

Study	Brorsson et al. [20]
**JBI**	9/13
**True randomisation used for assignment of participants to treatment groups**	YES
**Allocation to treatment groups concealed**	YES
**Treatment groups similar at the baseline**	YES
**Participants blinded to treatment allocation**	NO
**Those delivering treatment blinded to treatment allocation**	NO
**Outcomes assessors blinded to treatment allocation**	NO
**Treatment groups treated identically other than the intervention of interest**	YES
**Follow-up complete and if not, were differences between groups in terms of their follow-up adequately described and analysed?**	YES
**Participants analysed in the groups to which they were randomised**	NO
**Outcomes measured in the same way for treatment groups**	YES
**Outcomes measured in a reliable way**	YES
**Appropriate statistical analysis used**	YES
**Trial design appropriate for the topic, and any deviations from the standard RCT design accounted**	YES

Yes: ✓, No: ✗, Not clear or Not Applicable: N/A; JBI: Joanna Briggs Institute

Table S4. Scores of qualitative research.

Studies	JBI	Congruence between stated philosophical perspective and research methodology	Congruence between research methodology and research question/objectives	Congruence between research methodology and methods used to collect data	Congruence between research methodology and representation and analysis of data	Congruence between research methodology and interpretation of results	Cultural and theoretical localisation	Influence of the researcher on the research and vice versa is addressed	Representation of participants and their voices	Ethical approval by an appropriate body	Relationship between findings and data analysis or data interpretation
Commissariat et al. [23]	10/10	YES	YES	YES	YES	YES	YES	YES	YES	YES	YES
Jeong et al. [29]	10/10	YES	YES	YES	YES	YES	YES	YES	YES	YES	YES
Robertson et al. [36]	10/10	YES	YES	YES	YES	YES	YES	YES	YES	YES	YES
Sien et al. [38]	10/10	YES	YES	YES	YES	YES	YES	YES	YES	YES	YES

Note. Studies presented by authors' surname alphabetical order. JBI: Joanna Briggs Institute.
